# A morphological re-evaluation of *Pachyseiushumeralis* Berlese, 1910 (Acari, Mesostigmata, Pachylaelapidae)

**DOI:** 10.3897/zookeys.790.26894

**Published:** 2018-10-15

**Authors:** Peter Mašán

**Affiliations:** 1 Institute of Zoology, Slovak Academy of Sciences, Dúbravská cesta 9, 845-06 Bratislava, Slovakia Institute of Zoology, Slovak Academy of Sciences Bratislava Slovakia

**Keywords:** Description, morphology, soil mites, taxonomic revision, type species, systematics

## Abstract

Based on features of the lectotype and newly collected specimens from Italy (Boboli Gardens, Florence), a morphological concept of *Pachyseiushumeralis* Berlese, 1910 is revised and re-evaluated. New diagnostic character states important for recognition of the species are provided. A misidentified species, formerly widely published in Europe under the name *P.humeralis*, is established as a new species, *Pachyseiussubhumeralis***sp. n.**

## Introduction

*Pachyseiushumeralis* was quite briefly and insufficiently described by Berlese in 1910, as the type species of the genus *Pachyseius*, originally based on female specimens from the two collection sites in Italy (Rome, Mugello). Later in his next paper, Berlese (1913) supported his primary description of the species by adding of some illustrations (ventral idiosoma with gnathosoma, and tarsus II), based on the specimen from Rome (pers. obs.).

Several specimens of *Pachyseiushumeralis* are present in the Berlese Collection, Florence, all from several localities of Central Italy (Maccarese close to Rome, Monte Giovi in Mugello Region, Filettino in Lazio Region, Vallombrosa in Regello Municipality, and Boboli Gardens in Florence), but only two of them (numbered 83/5 and 83/6) belong to the original series of Berlese. In his original description of *P.humeralis*, Berlese (1910) did not designate a holotype, so his two specimens from “Roma” (83/5) and “Mugello” (83/6) must be considered as syntypes. Mašán (2007) based his revision of *P.humeralis* upon these two syntypes of which one of them, from Maccarese, Rome, is labelled “tipico” (83/5) by Berlese, and should be considered to be lectotype. Mašán (2007) found that the type specimens examined by him belong to two different species. The lectotype female mounted onto the slide 83/5 was incorrectly considered by him to be identical with the species which was redescribed and illustrated by several authors (Hyatt 1956, Karg 1971, Solomon 1982, Lapina 1988, Mašán 2007). Mašán (2007) overlooked specific morphological differences, described in present paper, between the lectotype and the specimens which were available for his comparative study from Slovakia and other countries in Europe. The paratype female in slide 83/6 was identified as *Pachyseiuswideventris* Afifi & Nasr, 1984 originally described from Netherlands (Afifi and Nasr 1984). The concept of *P.humeralis* is therefore based on a single specimen, from Rome.

In the Boboli Gardens in Florence, Italy, fourteen specimens of a species which was putatively considered as a new and closely related with *Pachyseiushumeralis*, were collected by me in leaf litter and soil detritus. In order to define the differences between the newly collected specimens and *P.humeralis*, the lectotype of the species was re-examined. The analysis indicated that my specimens from the Boboli Gardens in Florence were actually clearly conspecific with that on slide number 83/5 of Berlese. So now I can confirm my mistake, and reveal the true identity of *P.humeralis* sensu Berlese (1910).

The main aim of this study is to diagnose and redescribe the type species of *Pachyseius*, misinterpreted by the followers of Berlese (1910, 1913), and compare that species with similar but confused species widely reported from various European countries under the name *Pachyseiushumeralis*.

## Materials and methods

Collected mites were extracted from the litter and soil detritus by means of a modified Berlese-Tullgren funnel equipped with a 40-Watt bulb, and preserved in ethyl alcohol. Before identification, the mites were mounted onto permanent microscope slides, using Swan’s chloral hydrate mounting medium. A Leica DM 1000 light microscope equipped with a Leica EC3 digital camera was used to obtain measurements and photos. Some multiple images were combined using the CombineZP software program (Hadley 2010). Measurements were made from slide-mounted specimens. Lengths of shields and legs were measured along their midlines, and widths at their widest point (if not otherwise specified in the description). Dorsal setae were measured from the bases of their insertions to their tips. Measurements are mostly presented as ranges (minimum to maximum). The terminology of dorsal and ventral chaetotaxy follows Lindquist and Evans (1965), and that for leg setae follows that of Evans (1963). Identification of pore-like structures on the idiosomal integument is based on the morphological observations of Athias-Henriot (1969); notation for these structures such as adenotaxy and poroidotaxy follows Johnston and Moraza (1991).

For the purpose of this study, all the specimens from the large-scale collections so far reported from Slovakia under the confused name *Pachyseiushumeralis* by the author (see Mašán 2007) were checked once again for their correct identity. If available, also specimens recently collected in various European countries were re-examined and listed below.

## Systematics

### Pachyseius humeralis

Taxon classificationAnimaliaMesostigmataPachylaelapidae

Berlese, 1910

**Figures 1–2.**
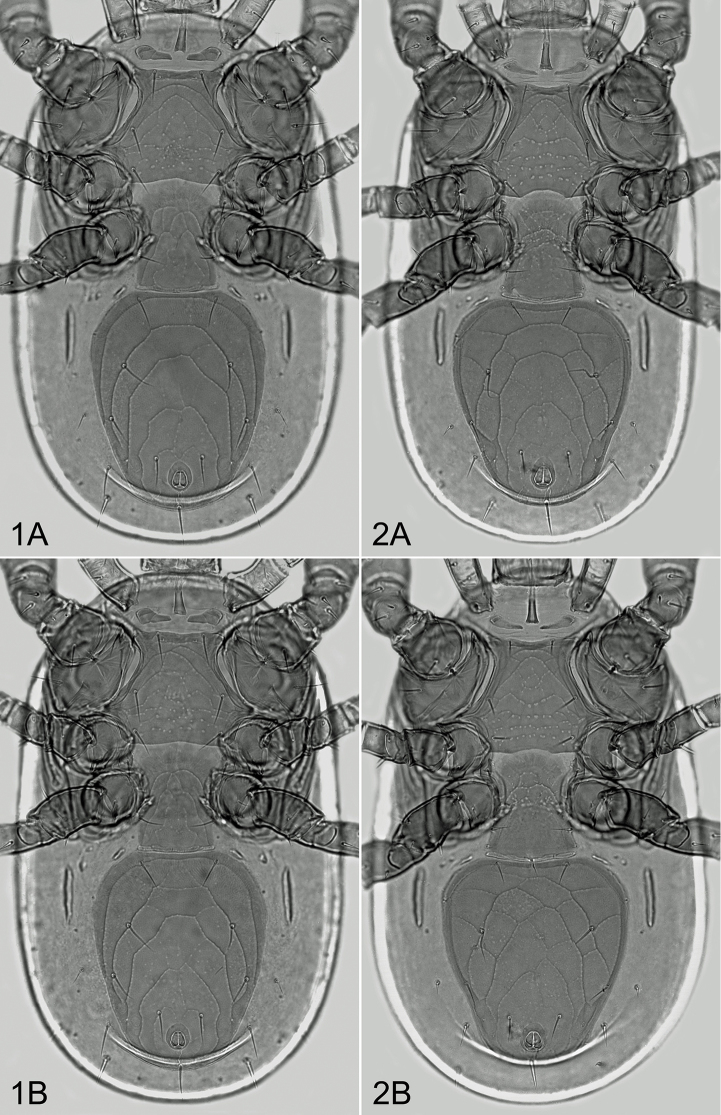
*Pachyseius* spp., ventral idiosoma of females. **1***Pachyseiushumeralis***2***Pachyseiussubhumeralis* sp. n. Not to scale **A, B** variant specimens.

**Figures 3–7.**
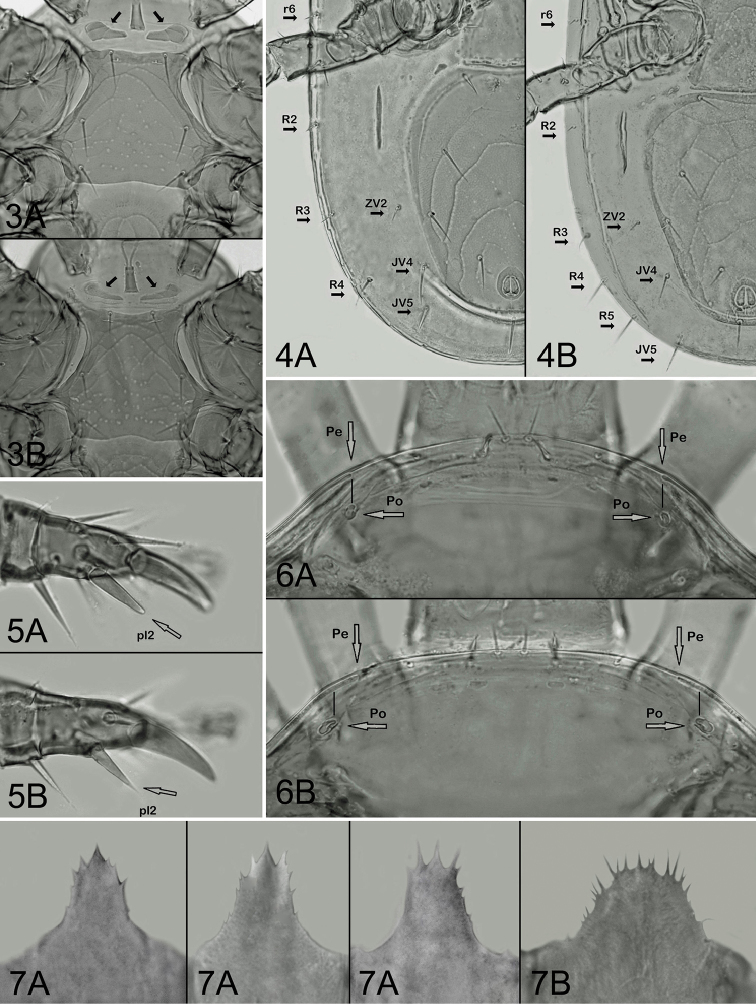
*Pachyseiushumeralis* (**A**) and *Pachyseiussubhumeralis* sp. n. (**B**), females. **3** Sternal regions (arrows pointing to presternal platelets) **4** Opisthogastric regions (arrows pointing to marginal and opisthogastric setae placed on soft integument) **5** Tarsi II (arrows pointing to seta pl2) **6** Anteriormost portion of dorsal shields [arrows pointing to anterior tip of peritremes (Pe) and enlarged gland pores gdj3 (Po)] **7** Epistomes (with three variant forms in *P.humeralis*). Not to scale.

Figures 1A , 1B , 3A , 4A , 5A , 6A , 7A

Pachyseius humeralis Berlese, 1910: 255; Berlese 1913: 81; Castagnoli and Pegazzano 1985: 187 (in part).
non Pachyseiushumeralis: Nefedov 1966: 1098 (= Pachyseiuswideventris Afifi & Nasr, 1984) (a newly introduced misidentification).

#### Material examined.

Lectotype by present designation: female (slide number 83/5), **Italy**, Maccarese Village (Rome), humus, labelled as *Pachylaelapshumeralis*, deposited at the Research Centre for Agrobiology and Pedology, Florence; other specimens: 14 females, **Italy**, Florence City, Boboli Gardens, leaf litter and soil detritus, May 21, 2006, leg. P. Mašán, deposited at the Institute of Zoology, Slovak Academy of Sciences, Bratislava.

#### Diagnosis.

The species may be distinguished from the other congeners especially by combination of the following female characters: (1) dorsal shield setae simple, needle-like; (2) dorsal shield between setae z1 and z2 and peritrematal shields close to stigma with enlarged and cavity-like poroid structure; (3) presternal platelets well sclerotized, with two striae, separate each other, and free from anterior margin of sternal shield; (4) exopodal platelets II–III and III–IV free, not fused to peritrematal shields; (5) ventrianal shield with three pairs of preanal setae (JV1‒JV3); (6) lateral and opisthogastric soft integument with seven pairs of setae: r6, R2‒R4, ZV2, JV4, and JV5; (7) tarsus II with two subdistal posterolateral setae thickened, spur-like; (8) tarsus IV with 17 setae.

#### Description.

*Female. Dorsal idiosoma*. Dorsal shield 540–600 μm long (most frequently 565–595 μm), 320–380 μm wide, suboval, oblong (length/width: 1.6–1.75), widely rounded anteriorly and posteriorly, with almost parallel lateral margins, and delicate reticulation on posterior surface. Dorsal shield with 30 pairs of setae; the setae simple, smooth, needle-like and mostly similar in length; the length of some selected setae as follows: z1 7–10 μm, j1 15–19 μm, j5 19–25 μm, J1 24–28 μm, J2 and J3 27–32 μm, J4 30–36 μm, J5 31–39 μm; the longest dorsal setae 40–48 μm in length. A pair of gland pores gdj3 enlarged, cavity-like, well sclerotized, and situated between setae z1 and z2 close to anterior margin of dorsal shield. Anterior surface with two pairs of minute suboval sclerites situated between setae j2 and j3.

*Ventral idiosoma* (Figure 1A, B). Presternal area with two platelets (Figure 3A); the platelets free on soft integument, small, subtriangular, with two striae transversely or obliquely oriented on their well sclerotized surface. Sternal shield 112–125 μm long, 76–87 μm wide at the narrowest level of coxae II (120–140 μm at the level of setae st2), with three pairs of subequal setae (st1–st3 36–42 μm), and two pairs of lyrifissures; the shield with anterior and posterior margin almost straight or very slightly concave; sculptural ornamentation well developed, with curved punctate lines on anterior and lateral surface and polygonal or linear pattern of punctations on medial and posterior surface. Two metasternal platelets suboval, each with a seta and pore-like structure. Epigynal shield 110–130 μm long, 74–90 μm wide, oblong, with convex anterior margin, truncate posterior margin, two genital setae, and delicate punctate-reticulate pattern on surface; a row of four suboval and elongate postgenital sclerites along its posterior margin present; genital pore-like structures normally situated on soft integument, outside the shield. Ventrianal shield suboval, longer than wide (length: 218–252 μm, width: 168–198 μm, length/width: 1.2–1.37), with anterior portion moderately expanded, widest anterior to setae JV2, straight or mostly slightly concave anteriorly, widely rounded anterolaterally and posteriorly, bearing lineate-reticulate pattern on entire surface (parallel lines more expressively indicated), three pairs of preanal setae (JV1–JV3), and three circum-anal setae close to suboval anus. Peritrematal shields free from closely adjacent exopodal platelets II–III and III–IV; post-stigmatic sections of the shields narrowed posteriorly, rounded terminally, not reaching beyond the posterior margin of exopodals III–IV, and each bearing three small pore-like structures and one greatly enlarged cavity-like poroid close to stigma. Peritremes normal, with anterior tips reaching marginal dorsal surface between setae z1 and z2, close to enlarged gland pores gdj3 (Figure 6A). Exopodal platelets III–IV free but contiguous to exopodals II–III. Soft integument between peritrematal shields and anterolateral margins of ventrianal shield with two pairs of minute irregular sclerites and a pair of larger suboval platelets. Metapodal region with a pair of narrow, conspicuously elongated and longitudinally oriented platelets; the platelets 52–69 μm long and relatively well separated from anterolateral margins of ventri-anal shield. Lateral and opisthogastric soft integument with seven pairs of setae (Figure 4A): four pairs of dorsomarginal (r6, R2–R4) and three pairs of opisthogastric setae (ZV2, JV4, JV5). All ventrally inserted setae similar to those on dorsal shield.

*Gnathosomal structures*. Corniculi slender and horn-like; deutosternal groove with four or five transverse rows of denticles and two smooth transverse lines; internal malae reaching beyond the corniculi; gnathosomal setae smooth and needle-like. Palptibia without outgrowths, palptarsus with three-tined apotele. Epistome narrow, with anterior and lateral margin irregularly dentate, apex with larger and pointed central cusp (Figure 7A).

*Legs.* Leg setation as previously described in the genus (Mašán 2007), and for *Pachyseiushumeralis* species group: tarsus IV bearing 17 setae (seta ad2 absent). Tarsus II with two obtuse spur-like distal setae pl1 and pl2 (Figure 5A).

*Sperm induction system.* Tubiform structures of sperm induction system detectable, weakly sclerotized, long and thin, and associated with posterior margin of coxae III.

### Pachyseius subhumeralis sp. n.

Taxon classificationAnimaliaMesostigmataPachylaelapidae

http://zoobank.org/3B4BDFFF-BE84-4FF9-B2E0-91E053317BC8

Figures 2A , 2B , 3B , 4B , 5B , 6B , 7B

Pachyseius humeralis : Hyatt 1956: 4; Karg 1971: 141; Solomon 1982: 102; Lapina 1988: 178; Karg 1993: 116; Mašán 2007: 32, 210.

#### Material examined.

***Type material.*** Holotype female: **Slovakia**, Trnavská Pahorkatina Highland, Štefanová Village, Dubník Forest, oak forest (with *Quercus* spp.), leaf litter and soil detritus, altitude 250 m, June 18, 1997, leg. P. Mašán. Paratypes: 2 females, with the same collection data as in holotype; seven females, **Slovakia**, Malé Karpaty Mts., Bratislava Capital, Železná Studienka Forest, beech forest (with *Fagussylvatica*), leaf litter and soil detritus, altitude 370 m, April 27, 1993, leg. P. Mašán; 17 females, Bratislava Capital, Devín Settlement, Devínska Kobyla Mt., oak forest (with *Quercus* spp.), leaf litter and soil detritus; altitude 270 m, July 30, 1997, leg. P. Mašán. All these specimens were previously published as *Pachyseiushumeralis* by Mašán (2007), and are deposited at the Institute of Zoology, Slovak Academy of Sciences, Bratislava.

***Non-type material.* Bulgaria**: one female, Shumen Plateau Natural Park, Shumen City, Bukaka Reserve, old beech forest (*Fagussylvatica*) with admixed hornbeam (*Carpinusbetulus*), leaf litter and soil detritus, altitude 500 m, October 23, 2007, leg. I. Mihál. **Czech Republic**: two females, with unknown collection data. **France**: two females, Alpes Cottiennes Mts., Arvieux Village, Gorges du Guil Canyon, pine forest (*Pinus* sp.) on a slope with loose rock debris (limestone), humid needle litter with tussocks of grass and mushrooms, altitude 1,180 m, June 11, 2007, leg. P. Fenďa. **Germany**: three females, Bavaria, Bavarian Prealps Mts., Flintsbach am Inn Village, St. Peter’s Abbey on the Madron (“Peterskirchlein”), broadleaved deciduous forest predominated by beech (*Fagussylvatica*), humid soil detritus under a deep layer of leaf-fall, altitude 600 m, April 25, 2007, leg. P. Mašán. **Hungary**: six females, with unknown collection data. **Italy**: four females, Lombardy Region, Bergamo Province, Bergamasque Alps and Prealps Mts., Zambla Alta Village, near to Zambla Pass, spruce forest (*Piceaabies*) with admixed beech (*Fagussylvatica*), needle litter and soil detritus with decomposed wood substrate, altitude 1,170 m, May 13, 2015, leg. P. Mašán; three females, Tuscany Region, Florence City, Villa Camerata Hostel, park, broadleaved deciduous wood, leaf litter, altitude 75 m, April 23, 2009, leg. P. Mašán; two females, Florence City, Parco delle Cascine Gardens, broad-leaved deciduous wood, leaf litter with soil and wood detritus, altitude 45 m, May 23, 2006, leg. P. Mašán. **Poland**: one female, with unknown collection data. **Romania**: one female, Transylvania Region, Apuseni Mts. (Gilău Mts. in Bihor Massif), Cluj County, Turda Town, Turda Gorge, near to Peştera Ungurească Cave, oak-hornbeam forest, leaf litter, altitude 530 m, July 13, 2006, leg. P. Fenďa. **Serbia**: three females, Kučajske Planine Mts., Pomoravlje District, Troglan Bara Settlement, Velika Brezovica Forest, beech forest (*Fagussylvatica*), leaf litter and soil detritus, altitude 900 m, April 23, 2009, leg. I. Mihál. **Switzerland**: four females, Basel-Stadt Canton, Riehen Town, Wenken Settlement, old beech forest with limes (*Tilia* sp.) and maples (*Acer* sp.), leaf litter and soil detritus, altitude 380 m, June 1, 2016, leg. P. Mašán. **United Kingdom, Wales**: two females, Anglesey Island, Llangefni Town, The Dingle Forest, alluvium of Cefni River, mixed broadleaved deciduous forest, leaf litter and soil detritus, altitude 35 m, July 31, 2010, leg. P. Mašán; four females, Anglesey Island, Llandegfan Village, alluvium of Cadnant River, mixed broadleaved deciduous wood, leaf litter and soil detritus, altitude 50 m, July 25, 2010, leg. P. Mašán.

#### Diagnosis.

The species may be distinguished from the other congeners especially by combination of the following female characters: (1) dorsal shield setae simple, needle-like; (2) dorsal shield between setae z1 and z2 and peritrematal shields close to stigma with enlarged and cavity-like poroid structure; (3) presternal platelets well sclerotized in anterior part, transversely striate, separate each other but connected to anterior margin of sternal shield; (4) exopodal platelets II-III and III-IV free, not fused to peritrematal shields; (5) ventrianal shield with three pairs of preanal setae (JV1‒JV3); (6) lateral and opisthogastric soft integument with eight pairs of setae: r6, R2‒R5, ZV2, JV4, and JV5; (7) tarsus II with two subdistal posterolateral setae thickened, of which pl1 with obtuse apex, spur-like, and pl2 terminally attenuate and sharply pointed (but the tip of pl2 is fragile and can be very often broken in slide-mounted specimens); (8) tarsus IV with 17 setae.

#### Description.

The morphological attributes of the species were described and illustrated in detail by Mašán (2007), and the description does not need to be repeated here. The original illustrations given by the cited author are based on the type specimens from Štefanová Village (see above).

#### Etymology.

Many epithets beginning with *sub*-, a Latin name-forming prefix meaning “approaching”, are intended to distinguish a species from that with which it was previously confused. The new epithet is proposed as an alternative name for *Pachyseiushumeralis* in the broad sense understood to date.

#### Discussion.

The re-evaluation of the lectotype and newly collected material of *Pachyseiushumeralis* sensu Berlese, 1910 showed that there are several apparent and constant morphological differences between this species and its congener widely reported from Europe under the same name, and here established as *Pachyseiussubhumeralis* sp. n. According to these findings, *P.humeralis* may be most reliably distinguished from *P.subhumeralis* by the following characters:

(1) the placement of genital pores (placed outside the epigynal shield in *P.humeralis*, or on posterolateral corners of the shield in *P.subhumeralis*; Figs 1 and 2),

(2) the form and placement of presternal platelets (the platelets evenly sclerotized and free on soft integument in *P.humeralis*, or separately connected to sternal shield with their weakly sclerotized posterior portions in *P.subhumeralis*; Figure 3),

(3) the number of dorsomarginal setae on soft integument (four pairs in *P.humeralis* ‒ setae R5 absent, or five pairs in *P.subhumeralis* ‒ setae R5 present; Figure 4),

(4) the form of posterolateral seta pl2 on tarsus II (pl2 robust, spur-like, and apically rounded in *P.humeralis*, or regularly tapered and apically pointed in *P.subhumeralis*; Figure 5),

(5) the relative length of peritremes (the peritreme with anterior tip never reaching beyond the longitudinal axis of gland pore gdj3 in *P.humeralis*, or reaching slightly beyond this point in *P.subhumeralis*; Figure 6),

(6) the form of epistome (the epistome with narrow base and large central cusp in *P.humeralis*, or with widened base and uniformly spinate anteriormost margin in *P.subhumeralis*; Figure 7).

There are also less serious differences recognizable between the two compared species, having partly transitional character, for instance in size and proportions of the dorsal and ventroanal shield, and relative length of some idiosomal setae. Generally, when compared with *Pachyseiussubhumeralis* (based on metric data given by Mašán in 2007), *Pachyseiushumeralis* is smaller species (optimum length: 565–595 μm versus 595–640 μm), possessing relatively narrower ventrianal shield (L/W ratio: 1.2‒1.37 versus 1.08‒1.28; Figs 1 and 2), relatively longer setae (j5: 19–25 μm versus 15–23 μm, J5: 31–39 μm versus 24–34 μm), and shallower medial concavity of posterior margin of sternal shield.

The specimens of *Pachyseiushumeralis* available in the Berlese Collection in Florence from several localities of Central Italy (Monte Giovi, Filettino, Vallombrosa, and Florence), and those from other European regions widely published by various authors (especially from the Mediterranean areas), should be carefully re-examined in the next studies to define their correct identity, and to re-evaluate spatial distribution of the species treated in this paper.

## Supplementary Material

XML Treatment for Pachyseius humeralis

XML Treatment for Pachyseius subhumeralis
